# An experimental study on the impacts of inspiratory and expiratory muscles activities during mechanical ventilation in ARDS animal model

**DOI:** 10.1038/srep42785

**Published:** 2017-02-23

**Authors:** Xianming Zhang, Juan Du, Weiliang Wu, Yongcheng Zhu, Ying Jiang, Rongchang Chen

**Affiliations:** 1 Department of Respiratory Medicine, First Affiliated Hospital of Guizhou Medical University, Guizhou, China; 2 Respiratory Mechanics Lab, State Key Laboratory of Respiratory Disease, Guangzhou Institute of Respiratory Disease, First Affiliated Hospital of Guangzhou Medical University, Guangzhou, Guangdong, China

## Abstract

In spite of intensive investigations, the role of spontaneous breathing (SB) activity in ARDS has not been well defined yet and little has been known about the different contribution of inspiratory or expiratory muscles activities during mechanical ventilation in patients with ARDS. In present study, oleic acid-induced beagle dogs’ ARDS models were employed and ventilated with the same level of mean airway pressure. Respiratory mechanics, lung volume, gas exchange and inflammatory cytokines were measured during mechanical ventilation, and lung injury was determined histologically. As a result, for the comparable ventilator setting, preserved inspiratory muscles activity groups resulted in higher end-expiratory lung volume (EELV) and oxygenation index. In addition, less lung damage scores and lower levels of system inflammatory cytokines were revealed after 8 h of ventilation. In comparison, preserved expiratory muscles activity groups resulted in lower EELV and oxygenation index. Moreover, higher lung injury scores and inflammatory cytokines levels were observed after 8 h of ventilation. Our findings suggest that the activity of inspiratory muscles has beneficial effects, whereas that of expiratory muscles exerts adverse effects during mechanical ventilation in ARDS animal model. Therefore, for mechanically ventilated patients with ARDS, the demands for deep sedation or paralysis might be replaced by the strategy of expiratory muscles paralysis through epidural anesthesia.

The mainstream supportive measure for patients suffering from acute respiratory distress syndrome (ARDS) is mechanical ventilation^1^. Despite being lifesaving, mechanical ventilation itself can lead to ventilator-induced lung injury (VILI)^2^, contributing to a high mortality^3^.

Mechanical ventilation methods for ARDS patients involve preserving spontaneous breathing (SB) or complete muscles paralysis^4^. In spite of intensive investigations, the role of SB activity in ARDS has not been well defined yet^5^. Many experimental and clinical studies have also reported that SB with inspiratory muscles activity, especially the diaphragm, can produce negative pleural pressures and transpulmonary pressure, which can improve ventilation distribution^6^, diminish atelectasis^7^, and thereby reduce mechanical stress and strain of lung^8^. It has been proved that preserving diaphragm activity in ventilated ARDS patients is correlated to fewer complications compared with muscles paralysis. The potential benefits include increasing the aeration of dependent lung areas^7^,^9^, promoting ventilation-perfusion matching^10^, improving global hemodynamics and organ perfusion^11^, decreasing the administration of drugs such as analgesic and sedative^12^, preventing ventilator-induced diaphragmatic dysfunction(VIDD)^13^,^14^, decreasing ventilator-induced lung injury (VILI)^15^,^16^ and so on. Thus, some investigators have claimed that SB should be preserved even in the most severe cases of ARDS^17^.

Nevertheless, little has been known about the effects of expiratory muscles activities during mechanical ventilation in patients with ARDS yet. During mechanical ventilation, expiration is a passive phenomenon generated by the elastic recoil forces of respiratory system. Nonetheless, an increased respiratory drive is prevalent in patients with ARDS. In the existence of an increased respiratory drive, SB with the activity of expiratory muscles, especially abdominal muscles, theoretically can increase positive pleural pressures and intra-abdominal pressure (IAP)^18^, which can decrease transpulmonary pressure, reduce the end-expiratory “baby lung” volume (EELV), and thereby lead to more alveolar collapse, lung consolidation and lung injury during mechanical ventilation^19^. Some studies have shown that the increase of IAP, even by 10 cmH_2_O, may worsen lung injury and cause organs dysfunction^20^,^21^. Prasad CV *et al*.^1^ revealed that the activation of abdominal muscles can impair pressure-controlled ventilation. A recent study has also demonstrated that the shear force produced by the alveolar opening and closing of lung increases the mortality in ARDS patients^22^,^23^.

In view of the advantages and disadvantages of SB during mechanical ventilation in patients with ARDS, it was hypothesized that the activity of inspiratory muscles had beneficial effects, while that of expiratory muscles had adverse effects. Consequently, the expiratory muscle of animal model was paralyzed through epidural anesthesia, and inspiratory muscle through phrenic nerve paralysis, to establish a model maintaining diaphragm (inspiratory muscle) activity and one preserving abdominal muscles (expiratory muscle) respectively. The aim was to explore the impacts and mechanism of inspiratory and expiratory muscles activities during mechanical ventilation in ARDS animal model and test the hypothesis that the demands for deep sedation or paralysis might be replaced by the strategy of expiratory muscles paralysis through epidural anesthesia.

## Materials and Methods

This study was approved by the Ethics Committee of Guizhou Medical University. The treatment and care of animals were in accordance with the standards of the university.

### Preparation of Animal samples

A total of 24 healthy beagle dogs (9.8–14.5 kg) were studied in the supine position. Anesthesia was completed by using Ketamine and continuous injection of Profocol. Paralysis was achieved with pancuronium. After orotracheal intubation with an 8.0-mm ID cuff tube, animals were ventilated with an EVITA 4 ventilator (Dräger Medical AG, Lübeck Germany). IPPV ventilation was set on at a VT of 10 ml/kg, FiO_2_ 1.0, PEEP 5 cm H_2_O, and I: E ratio of 1:1. The respiratory rate (RR) was adjusted to keep PaCO_2_ within 35~45 mmHg. Lactated Ringer’s injection (6 ml/kg/h) was administered for hemodynamic stability. Catheters were inserted into the femoral artery and right jugular vein, and then connected to PiCCO system to measure mean arterial blood pressure (MPA), cardiac output and body temperature. Arterial blood samples were obtained using catheter and analyzed immediately.

Airway pressure (Paw), esophageal pressure (Peso) and intragastric pressure (Pgas) were recorded by using a multi-pair esophageal electrode-balloon combined catheter placed into the esophagus, the position of which was optimized with occlusion technique^24^. Airflow was measured by respiratory flow head, and integrated to obtain tidal volume. Powerlab 16/30 SP and Labchart 7.2 software on Macbook were applied to record the signals of Paw, Peso, Pgas, airflow, abdominal muscles surface electromyography (EMGab) and diaphragmatic esophageal surface electromyography (EMGdi). Animals’ body temperature was maintained at 37 °C with a heating pad, and averaged over eight breaths to calculate pressures, tidal volume, and respiratory rate.

### Experimental Protocol

After 30 min of stabilization and measurements at baseline, lung injury model was achieved through intravenous injection of 0.3 ml/kg purified oleic. If needed, additional infusion oleic acid (0.2 ml each time) would be given until PaO_2_/FiO_2_ became less than 100 mmHg. When the PaO_2_/FiO_2_ ratio were consistently below 100 mmHg for 30 min, a stable model of severe ARDS was considered to be established successfully^25^,^26^,^27^.

After the establishment of ARDS model and collection of data, the ventilator was switched to BIPAP mode, then the animals were randomly classified into four groups: (1) Spontaneous breathing group (BIPAP_SB,_ n = 6), both inspiratory and expiratory muscles activities were preserved; (2) Complete muscle paralysis group (BIPAP_PC,_ n = 6), treated with neuromuscular blocking agent (Pipecuronium bromide of 0.08 mg/kg): both inspiratory and expiratory muscles activities were absent; (3) Inspiratory muscles activity group (BIPAP_AI_, n = 6), treated with lumbar epidural anesthesia (ropivacaine hydrochloride at a speed of 1–2 ml/h for 8 h): inspiratory activities was preserved but expiratory muscles activities was absent; (4) Expiratory muscles activity group (BIPAP_AE,_ n = 6), treated with phrenic nerve transection: inspiratory activities was absent but expiratory muscles activities was preserved.

For BIPAP_PC_ group, P_high_ was titrated to achieve VT ≈ 6 ml/kg. P_low_ was set at 10 cmH_2_O, FiO_2_ 1.0, and fixed I: E = 1:1 to minimize mean Paw changes. Mandatory RR was regulated to maintain PaCO_2_ within 35 to 60 mmHg. For BIPAP_SB_ group, the infusion of pancuronium was stopped to recover SB, and other ventilator settings were the same as those of BIPAP_PC_ group. SB was confirmed by the negative deflection of Peso. For BIPAP_AI_ group, the method of paralyzing abdominal muscles was similar to that described by Warner DO^28^. An epidural catheter was inserted via the second tail vertebra, and its tip was pushed forward to the position close to L_4_ or L_5_ lumbar vertebrae in the epidural space confirmed by visual observation or autopsy. 2% lidocaine was injected slowly into incremental doses of 0.5 ml via the epidural catheter until the EMGab was abolished. The subsequent continuous infusion of ropivacaine at a speed of 1–2 ml/h and other ventilator settings were the same as those of BIPAP_SB_ group. As for BIPAP_AE_ group, preserving expiratory muscles activity alone was achieved through phrenic nerve transection, and other ventilator settings were the same as those of BIPAP_SB_ group.

All measurements were performed every 2 hours. P_L_ were calculated by the difference between Paw and Peso. During BIPAP ventilation mode, mean Paw can be calculated as follows^29^,^30^: (P_high_ × T_high_ + P_low_ × T_low_)/(T_high_ + T_low_), where T_high_ is the length of time for P_high_, and T_low_ is that for P_low_. When RR was adjusted to fix T_high_: T_low_ ratio at 1:1, mean Paw could keep constant at (P_high_ + P_low_)/2. With the above method, the mean Paw of all experimental groups was maintained the same, regardless of the existence of SB. A simplified closed-circuit helium dilution method was utilized to measure EELV at P_low_10 cmH_2_O during an end-expiratory pause^31^. Dead space/tidal volume ratio (VD/VT) was calculated by using Enghoff equation^32^. Samples of IL-6 and IL-8 in plasma were collected before and after the induction of lung injury at the end of the 8 h of MV. Supernatant aliquots were frozen at −80 °C for analysis after being centrifuged at 3,000 rpm for 15 min. An ELISA kit for dogs was employed to measure the Plasma levels of IL-6 and IL-8^30^. After eight hours of ventilation, the animals were euthanized with 20 ml of intravenous 10% potassium chloride. Five sections in the right upper, middle and lower lobes were stained with hematoxylin and eosin for pathological analysis. Lung tissue was examined by a pathologist blinded to the group allocations. Based on combined pathomorphological changes criteria, lung injury severity was rated on a five-point scale, involving alveolar congestion, alveolar edema and interstitial edema, lymphocytes infiltration, erythrocytes infiltration and granulocytes infiltration, micro thrombi as well as fibrinous exudates. Each sample was graded as follows^15^,^33^: minimal changes: 0; mild: 1, moderate: 2; severe: 3; maximal changes: 4. The sum of graded scores was the total histopathological lung injury score.

### Statistical Analysis

All data are represented as means ± SD. Kolmogorov–Smirnov test was adopted to assess normal distribution. Paired t-test was utilized to compare the continuous data of the same group before and after the interventions. Multiple-group comparisons were made through ANOVA or Kruskal-Wallis test as appropriate. Repeated measures ANOVA were applied to test respiratory variables changes between different time points and groups, and a post hoc analysis was performed following LSD-t procedure as appropriate. IBM SPSS Statistics 21 was used for statistical analyses, and *P* < 0.05 was considered to be statistical significance of difference.

## Results

In fact, a total of 27 beagle dogs were employed, and 24 of them finished the experiment. At baseline, no significant differences were observed in HR, MPA, OI and respiratory mechanics parameters. After inducing injury, the gas exchange worsened, and the values of OI decreased below100 mmHg. Besides, significant differences were observed compared with the values of OI at baseline in all experimental groups.

**Figure 1:**
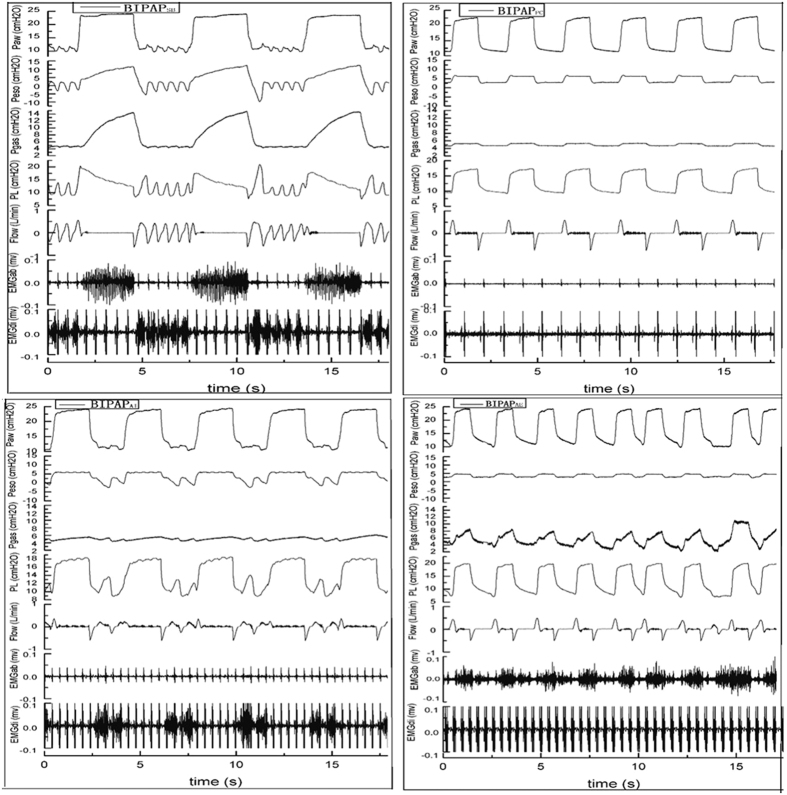
Representative respiratory tracings of airway pressure (Paw), esophageal pressure (Pes), intragastric pressure (Pgas), transpumonary pressure (PL), Airflow, abdominal muscles surface electromyography (EMGab) and diaphragmatic esophageal surface electromyography (EMGdi) in BIPAP_SB_, BIPAP_PC_, BIPAP_AI_ and BIPAP_AE_ group in representative animals. BIPAP_SB_ = biphasic positive airway pressure with SB; BIPAP_PC_ = biphasic positive airway pressure with muscles paralysis; BIPAP_AI_ = biphasic positive airway pressure with inspiratory muscles activity; BIPAP_AE_ = biphasic positive airway pressure with expiratory muscles activity.

Figure 1 shows the tracing records of Paw, Pes, Pgas, P_L_, Airflow, EMGab and EMGdi in four groups of representative animals. The mean Paws were comparable for all groups during the entire experiment. SB occurred rarely at P_high_ in all groups. Due to its preserving of both inspiratory and expiratory muscles activities, BIPAP_SB_ group presented larger fluctuations of Pes, Pgas and peak P_L_ compared with other groups, and kept the ratio of SB to total MV above 60%. In BIPAP_PC_ group, no inspiratory nor expiratory muscles activity was observed, so its Peso showed a positive change in the inspiratory phase. In BIPAP_AI_ group, which showed only inspiratory muscles activity, presented lower ΔPeso, Pgas, peak P_L_, more even P_L_ and longer time at P_high_ compared with BIPAP_SB_ group, and the ratio of SB to total MV decreased from 60%~100% to 10%~50%. In BIPAP_AE_ group, Peso showed no negative swing and was kept in positive range in the inspiratory phase.

**Figure 2:**
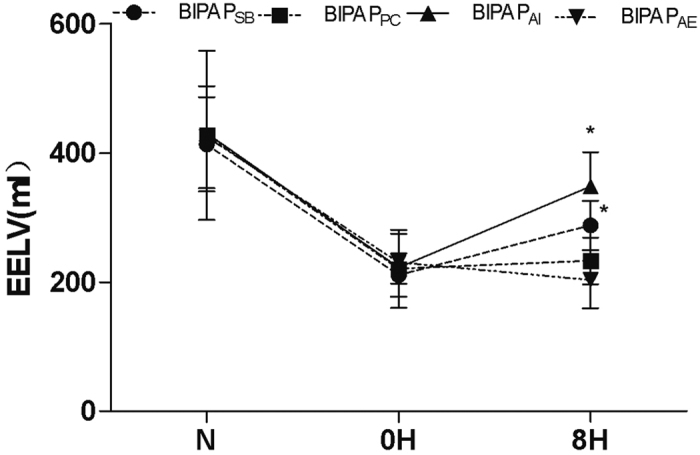
Time course of the dead space volume to tidal volume (VD/VT) ratio in experimental groups (n = 6 per group). BIPAP_SB_ = biphasic positive airway pressure with SB; BIPAP_PC_ = biphasic positive airway pressure with muscles paralysis; BIPAP_AI_ = biphasic positive airway pressure with inspiratory muscles activity; BIPAP_AE_ = biphasic positive airway pressure with expiratory muscles activity. SB = spontaneous breathing; *P < 0.05, vs. other groups.

**Figure 3:**
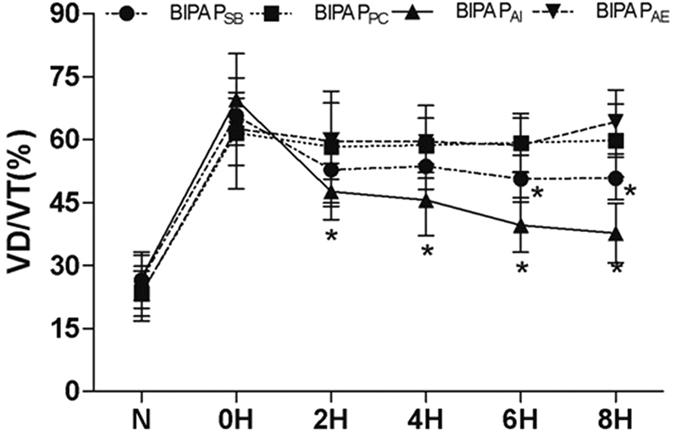
Time course of the end- expiratory lung volume (EELV) in experimental groups (n = 6 per group). BIPAP_SB_ = biphasic positive airway pressure with SB; BIPAP_PC_ = biphasic positive airway pressure with muscles paralysis; BIPAP_AI_ = biphasic positive airway pressure with inspiratory muscles activity; BIPAP_AE_ = biphasic positive airway pressure with expiratory muscles activity. SB = spontaneous breathing; *P < 0.05, vs. other groups.

As shown in Table 1, MPAs were similar among the groups during the entire experiment. The levels of PaCO_2_ were below 60 mmHg in all animals. However, BIPAP_AI_ and BIPAP_SB_ groups, which preserved inspiratory muscles activity presented with higher EELV than BIPAP_PC_ and BIPAP_AE_ groups respectively (*p* < 0.05) (Fig. 2). In addition, BIPAP_AI_ group resulted in a lower VD/VT compared with the other three groups after 2 h of ventilation (*p *<* *0.05). The VD/VT in BIPAP_SB_ group tended to be lower than those in BIPAP_PC_ and BIPAP_AE_ groups after 2 h of ventilation, and reached significant differences after 6 h of ventilation (*p *<* *0.05) (Fig. 3). BIPAP_AI_ group resulted in a higher OI compared with the other three groups after 2 h of mechanical ventilation. BIPAP_SB_ group presented a higher OI than BIPAP_PC_ and BIPAP_AE_ groups did after 6 h of ventilation (*p *<* *0.05).

**Figure 4:**
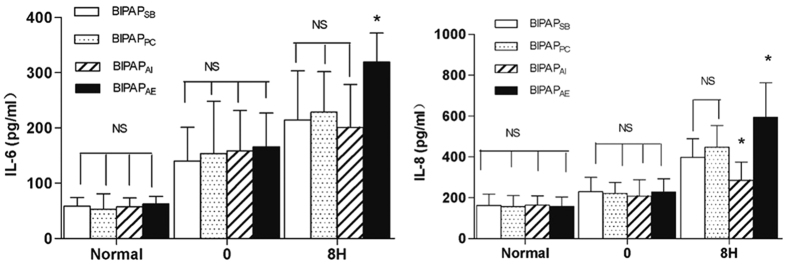
The Levels of interleukin (IL)-6 and IL-8 in plasma after 8 h mechanical ventilation. BIPAP_SB_ = biphasic positive airway pressure with SB; BIPAP_PC_ = biphasic positive airway pressure with muscles paralysis; BIPAP_AI_ = biphasic positive airway pressure with inspiratory muscles activity; BIPAP_AE_ = biphasic positive airway pressure with expiratory muscles activity. SB = spontaneous breathing; NS = no significantly difference. *P < 0.05, vs. other groups.

As indicated in Fig. 4: Plasma levels of IL-6 and IL-8 were comparable among groups before and after the induction of lung injury. After 8 h of MV, the lowest IL-8 levels was observed in BIPAP_AI_ group, and the highest IL-6 and IL-8 levels in plasma was observed in BIPAP_AE_ group when compared with other groups (*p* < 0.05).

**Figure 5:**
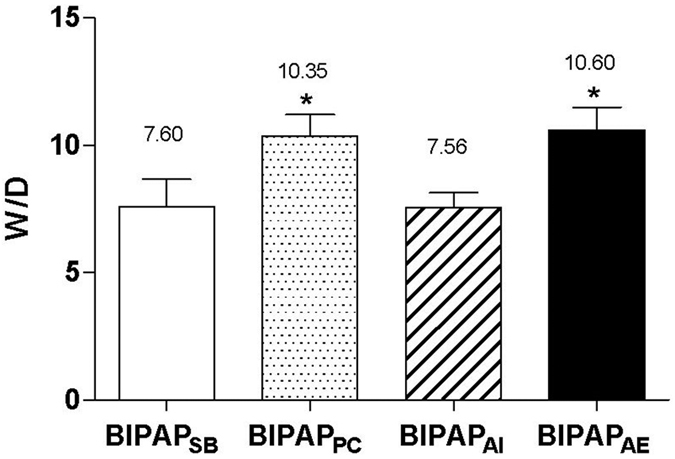
The Levels of Wet to dry weight ratio (W/D) after 8 h mechanical ventilation. BIPAP_SB_ = biphasic positive airway pressure with SB; BIPAP_PC_ = biphasic positive airway pressure with muscles paralysis; BIPAP_AI_ = biphasic positive airway pressure with inspiratory muscles activity; BIPAP_AE_ = biphasic positive airway pressure with expiratory muscles activity. SB = spontaneous breathing; NS = no significantly difference, *P < 0.05 vs. BIPAP_SB_ and BIPAP_AI_ groups.

**Figure 6:**
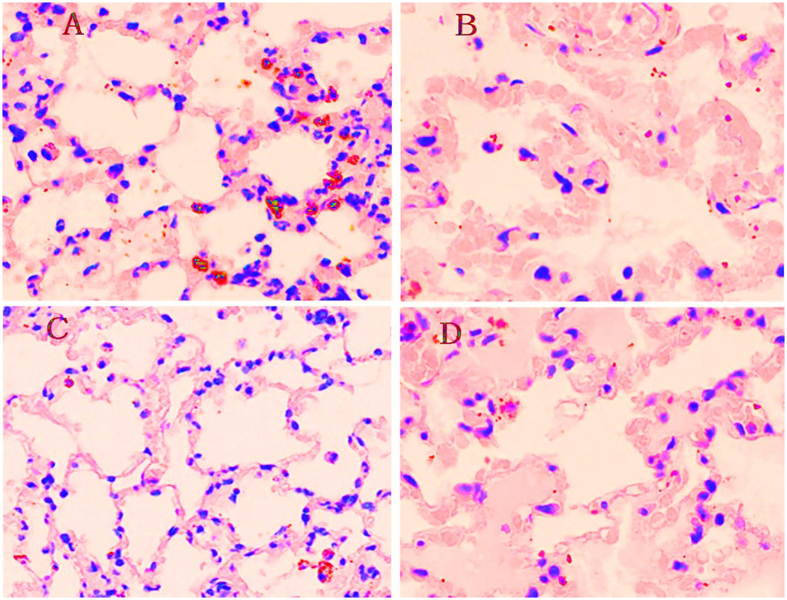
Representative appearances and photomicrographs of hematoxylineosin–stained lung sections (magnification × 200) from in BIPAP_SB_ (A), BIPAP_PC_ (B), BIPAP_AI_ (C) and BIPAP_AE_ (D) group in representative animals. BIPAP_SB_ = biphasic positive airway pressure with SB; BIPAP_PC_ = biphasic positive airway pressure with muscles paralysis; BIPAP_AI_ = biphasic positive airway pressure with inspiratory muscles activity; BIPAP_AE_ = biphasic positive airway pressure with expiratory muscles activity. The BIPAP_AI_ group had minimal alveolar congestion, and inflammatory cell infiltration. The BIPAP_AI_ group showed mild thickening of the alveolar walls, alveolar congestion, and hemorrhage. In the BIPAP_AE_ group, inflammatory cell infiltration, thickening of the alveolar walls, alveolar congestion, and more prominent hemorrhagic areas were observed.

As displayed in Table 2, BIPAP_AI_ and BIPAP_SB_ groups, which preserved inspiratory muscles activity, resulted in a lower sum of lung injury scores and wet/dry weight ratio (Fig. 5) in lung tissues compared with BIPAP_PC_ and BIPAP_AE_ groups (*p *<* *0.05). BIPAP_AI_ group presented less lung congestion, alveolar edema, alveolar infiltration of neutrophils and interstitial infiltration of lymphocyte. BIPAP_AE_ group showed more alveolar collapse, inflammatory cell infiltration, alveolar congestion, greater thickness of alveolar wall, and interstitial edema with hyaline membrane formation (Fig. 6).

## Discussion

On the basis of the ARDS animal model, the research findings indicate that the activation of inspiratory muscles increased EELV, improved oxygenation and decreased lung injury scores. On the contrary, the activation of expiratory muscles decreased EELV, worsened oxygenation and increased lung injury scores. That is to say, inspiratory and expiratory muscles had different impacts on ARDS animal model during mechanical ventilation. The activation of inspiratory muscles (diaphragm) had beneficial effects, while that of expiratory muscles (abdominal muscle) exerted adverse effects. Before discussing the results of this experiment, the following items need to be explained. An oleic acid-induced ARDS model with many basic features of ARDS was utilized in this study^27^. Treatment with a same dose of oleic acid in the same way can produce a reasonable reproducibility of lung damage^34^. Studies have confirmed an inverse correlation between injurious ventilation and IL-6, IL-8 levels. Hence, we selected IL-6 and IL-8, the most significant inflammatory factors during the mechanical ventilation in ARDS^35^. A static pressure–volume curve obtained through super syringe method showed that the lower inflection points were around 8–9 cm H_2_O for injury lungs. In consequence, P_low_ (PEEP) was set at 10 cm H_2_O for all experimental animals during mechanical ventilation.

To our knowledge, none of the previous studies has tried to separate the activities of inspiratory and expiratory muscles activities and explored the impacts of inspiratory and expiratory muscles activity during mechanical ventilation in ARDS. With a comparable ventilator setting, this study has proved that the activation of inspiratory muscles could lead to better oxygenation. This outcome can be easily explained. Firstly, inspiratory muscles activity increased EELV in this experiment. It has been proved that an increase in EELV is equivalent to the increase in oxygenation; secondly, it was also observed that inspiratory muscles activity reduced the VD/VT, which has a positive impact on oxygenation. Finally, inspiratory muscles activity improved oxygenation by promoting dorsal-caudal distribution of ventilation, and improving dead space ventilation and ventilation-perfusion matching.

Based on the findings of this research, the total lung injury score, wet/dry weight ratio in lung tissues as well as IL-6 and IL-8 levels in plasma were lower in BIPAP_AI_ groups. This outcome is similar to those of other studies with mild or moderate ARDS models^15^,^16^. From the represented tracing in the experiment, it could be observed that inspiratory muscles activity resulted in increased transpulmonary pressure at P_low_ (PEEP) without increasing transpulmonary pressure at P_high_. Increased transpulmonary pressure at P_low_ recruited collapsed lung units and favored more aeration into dependent regions, while increased EELV improved lung mechanical stress distribution, and reduced stress and strain (VT/EELV), as well as the major determinant of VILI. Furthermore, more aeration into dependent regions attenuated lung tissue recruitment and decruitment cycling, decreased hyperinflation in non-dependent lung zones, and thereby reduced lung injury.

In contrary to inspiratory muscles, the findings suggest that the activation of expiratory muscles worsen oxygenation. Douglas *et al*. have proved that EELV is parallel to oxygenation^36^. In this experiment, lower oxygenation was observed in BIPAP_AE_ groups as expiratory muscles activity decreased the EELV. It was also observed that expiratory muscles activity resulted in an increase of PTP, which means the work of breathing and oxygen consumption increased. In addition, the activation of expiratory muscles elevated IAP, decreased P_L_, reduced lung volume and increased compression atelectasis or consolidation^37^. The above factors led to a greater dead space and a higher heterogeneity of ventilation-perfusion ratio^38^, and thereby worsen gas exchange.

In patients with ARDS, the relationship between expiratory muscles activity and VILI is not clear. Henzler D *et al*.^20^ have proven that respiratory muscles activity during mechanical ventilation would cause greater lung damage in the presence of IAP. This study has also demonstrated that expiratory muscles activity would increase the W/D ratio, total lung injury scores and system inflammation. The potential mechanisms are as followings: Firstly, expiratory muscles activity could increase the value of ΔPes, which can promote the formation of pulmonary edema and aggravate lung injury^37^. Secondly, the activation of expiratory muscles could significantly increase Pgas, a surrogate of IAP. The activation of expiratory muscles, particularly abdominal muscles, can raise IAP even higher than 20 cm H_2_O^39^. Hence, the unopposed increase of IAP can cause greater lung injury by reducing P_L_ in dependent zones^20^. Thirdly, the activation of expiratory muscles could counteract the effect of PEEP of recruiting the collapsed lung, which would result in atelectrauma. Moreover, it was observed that the inactivation of expiratory muscles resulted in more even P_L_ and prolonged T_high_ which was presumed to achieve the aim of therapy for alveolar recruitment and attenuate lung injury; Reducing the high ratio of SB to total MV to 10~30% as clinically recommended during BIPAP mode of ventilation could decrease peak P_L_ and attenuate lung injury^7^. Finally, it was also observed the activation of expiratory muscles resulted in the reduction of EELV, so atelectrauma, lung strain, and main determinants of VILI may be further increased.

The current study has several major limitations. Firstly, BIPAP ventilated mode was used in this study. Therefore, we are not sure whether these results can be extended to other modes. Secondly due to protective strategy with a LTV used in this experiment, we cannot preclude the opposite effects of inspiratory or expiratory muscles activities on a high tidal volume injurious ventilation; Thirdly, the RR and nervous distribution of canine may not be the same as those of human beings. In view of this, it cannot be guaranteed that the the results of this study would be applicable to human patients and further studies are needed. Fourthly, an oleic acid-induced ARDS model was applied in this study, and its findings cannot be extrapolated to other ARDS models. Fifthly, since the long duration of ventilation time may influence the accuracy of the experiment, such as hypercapnia, influence of experimental procedure, and excessive use of drugs, observation of 8 hours of ventilation was used in this study. Indeed, a more prolonged study period might generate greater physiologic and morphologic difference between the experimental groups. Sixthly, in allusion to BIPAP_AP_ and BIPAP_PC_ groups, ropivacaine hydrochloride was adopted for paralysis, and propofol for anesthesia. Given this, the possibility that these drugs could affect pulmonary inflammatory response cannot be ruled out.

In conclusion, inspiratory and expiratory muscles in this animal model of ARDS have different impacts during mechanical ventilation. The activity of inspiratory muscles has beneficial effects, whilst that of expiratory muscles exerts adverse effects. As a result, the demands for deep sedation or paralysis might be replaced by the strategy of expiratory muscles paralysis through epidural anesthesia in mechanically ventilated patients with ARDS, which could preserve the advantages and avoid the disadvantages of SB. Nonetheless, changes in the management of mechanical ventilation in patients with ARDS require more evidence and a further research is necessary to confirm these results.

## Additional Information

**How to cite this article**: Zhang, X. *et al*. An experimental study on the impacts of inspiratory and expiratory muscles activities during mechanical ventilation in ARDS animal model. *Sci. Rep.* **7**, 42785; doi: 10.1038/srep42785 (2017).

**Publisher's note:** Springer Nature remains neutral with regard to jurisdictional claims in published maps and institutional affiliations.

## Supplementary Material

Supplementary Information

## Figures and Tables

**Table 1:** Hemodynamics and Respiratory Measurements.

Variables	Group (n = 6)	Basine	After Induction of ARDS					Time *Group Effect	Group Effect
			Injury	2 h	4 h	6 h	8 h		
MAP (mmHg)	**BIPAP**_**SB**_	115.6 ± 10.0	111.7 ± 14.6	113.2 ± 10.3	113.6 ± 10.1	107.7 ± 11.2	125.3 ± 16.1	0.325	0.743
	**BIPAP**_**PC**_	109.2 ± 10.9	116.8 ± 14.9	109.6 ± 11.9	114.2 ± 11.7	117.1 ± 14.8	114.4 ± 14.7		
	**BIPAP**_**AI**_	114.0 ± 5.8	127.2 ± 10.4	111.8 ± 12.1	113.7 ± 14.8	122.0 ± 12.3	111.7 ± 11.1		
	**BIPAP**_**AE**_	112.3 ± 14.9	109.7 ± 10.6	113.5 ± 15.3	113.6 ± 10.1	115.7 ± 14.2	116.3 ± 10.9		
HR (beats/min)	**BIPAP**_**SB**_	141 ± 16	127 ± 20	133 ± 15	118 ± 23	121 ± 19	128 ± 17	0.847	0.323
	**BIPAP**_**PC**_	137 ± 13	134 ± 25	131 ± 11	122 ± 20	126 ± 18	121 ± 7		
	**BIPAP**_**AI**_	142 ± 10	127 ± 22	133 ± 12	116 ± 16	120 ± 18	129 ± 14		
	**BIPAP**_**AE**_	126 ± 16	133 ± 13	126 ± 16	124 ± 11	125 ± 14	130 ± 12		
Total RR	**BIPAP**_**SB**_	24 ± 9	35 ± 3^b,d^	38 ± 12^b,d^	39 ± 12^b,d^	36 ± 6^b,d^	35 ± 12^b,d^	0.478	0.02
(breaths/min)	**BIPAP**_**PC**_	22 ± 7	44 ± 7^a,c^	46 ± 9^a,c^	41 ± 11^a,c^	47 ± 5^a,c^	47 ± 10^a,c^		
	**BIPAP**_**AI**_	21 ± 8	35 ± 8^b,d^	37 ± 6^b,d^	36 ± 11	38 ± 6^b,d^	36 ± 9^b,d^		
	**BIPAP**_**AE**_	23 ± 6	46 ± 15^a,c^	49 ± 9^a,c^	45 ± 6^a,c^	47 ± 11^a,c^	46 ± 11^a,c^		
VTave (ml/kg)	**BIPAP**_**SB**_	10.2 ± 0.3	6.4 ± 1.4	6.7 ± 1.4	6.4 ± 2.1	6.7 ± 1.5	6.5 ± 1.6	0.342	0.213
	**BIPAP**_**PC**_	10.1 ± 0.2	6.7 ± 0.9	6.7 ± 0.6	7.0 ± 0.7	7.1 ± 0.8	7.1 ± 0.7		
	**BIPAP**_**AI**_	9. 9 ± 0.3	6.4 ± 1.4	6.2 ± 0.8	6.6 ± 0.7	6.2 ± 0.7	6.5 ± 0.8		
	**BIPAP**_**AE**_	10.0 ± 0.3	7.0 ± 0.9	6.8 ± 0.6	7.0 ± 0.6	6.7 ± 0.8	6.8 ± 0.5		
MVtot (L/min)	**BIPAP**_**SB**_	2.9 ± 0.9	3.6 ± 1.4	3.7 ± 1.2	3.9 ± 1.8	3.3 ± 0.8^b,d^	3.5 ± 0.6^b,d^	0.542	0.473
	**BIPAP**_**PC**_	2.8 ± 0.9	4.3 ± 1.0	4.3 ± 1.1	4.5 ± 1.1	4.5 ± 0.7^a,c^	4.4 ± 0.5^a,c^		
	**BIPAP**_**AI**_	2.8 ± 1.1	3.9 ± 1.9	3.5 ± 1.2	3.8 ± 1.3	3.2 ± 0.9^b,d^	3.6 ± 0.7^b,d^		
	**BIPAP**_**AE**_	2.8 ± 0.9	4.5 ± 0.9	4.1 ± 1.4	4.5 ± 1.2	4.7 ± 0.9^a,c^	4.3 ± 0.6^a,c^		
PTP ml	**BIPAP**_**SB**_	—	—	87.9 ± 45^e^	95.9 ± 37^e^	87.9 ± 39^e^	92.7 ± 41^e^	0.528	0.008
	**BIPAP**_**PC**_	—	—	—	—	—	—		
	**BIPAP**_**AI**_	—	—	39.8 ± 19.5^e^	54.4 ± 22.7^e^	45.4 ± 26.3^e^	42.1 ± 19.8^e^		
	**BIPAP**_**AE**_	—	—	11.8 ± 9.5^e^	14.4 ± 12.3^e^	15.4 ± 10.6^e^	11.1 ± 9.0^e^		
PaO_2_/FiO_2_ (mmHg)	**BIPAP**_**SB**_	418 ± 34	84 ± 19	131 ± 24	171 ± 26^b,d^	197 ± 32^b,d^	231 ± 28^b,d^	0.015	0.032
	**BIPAP**_**PC**_	412 ± 29	86 ± 12	115 ± 26^c^	160. ± 35^c^	174 ± 49^a,c^	178 ± 39^a,c^		
	**BIPAP**_**AI**_	407 ± 33	82 ± 14	155 ± 27^e^	209 ± 30^e^	268 ± 49^e^	299 ± 36^e^		
	**BIPAP**_**AE**_	437 ± 37	90 ± 14	129 ± 53	133 ± 31	169 ± 27^a,c^	162 ± 51^a,c^		
PaCO_2_ (mmHg)	**BIPAP**_**SB**_	45 ± 5	54 ± 15	52 ± 8	54 ± 9	53 ± 5	55 ± 15	0.556	0.694
	**BIPAP**_**PC**_	43 ± 7	54 ± 10	50 ± 11	58 ± 9	53 ± 6	53 ± 4		
	**BIPAP**_**AI**_	42 ± 4	56 ± 18	58 ± 12	59 ± 4	59 ± 10	59 ± 11		
	**BIPAP**_**AE**_	42 ± 5	58 ± 8	58 ± 7	55 ± 5	54 ± 5	55 ± 6		
P_plat_ (cmH_2_O)	**BIPAP**_**SB**_	10.0 ± 1.0	22.5 ± 2.6	22.6 ± 2.3	22.5 ± 2.8	22.0 ± 2.2	22.5 ± 1.9	0.421	0.356
	**BIPAP**_**PC**_	10.0 ± 1.3	22.7 ± 2.8	21.4 ± 1.9	22.3 ± 2.6	21.6 ± 1.6	21.2 ± 2.7		
	**BIPAP**_**AI**_	9.5 ± 1.8	21.7 ± 1.0	22.4 ± 1.7	22.0 ± 2.2	22.1 ± 2.3	21.8 ± 2.8		
	**BIPAP**_**AE**_	9.5 ± 1.7	22.7 ± 1.4	22.7 ± 2.0	21.7 ± 2.8	21.3 ± 2.9	22.1 ± 2.5		
Mean Paw (cmH_2_O)	**BIPAP**_**SB**_	7.9 ± 0.8	17.7 ± 1.1	17.8 ± 1.2	18.1 ± 1.3	17.6 ± 1.4	17.2 ± 1.1	0.722	0.612
	**BIPAP**_**PC**_	7.3 ± 0.4	17.8 ± 1.1	17.9 ± 1.4	17.3 ± 1.2	17.7 ± 1.1	17.6 ± 0.9		
	**BIPAP**_**AI**_	7.6 ± 0.9	18.1 ± 1.2	17.9 ± 0.8	18.1 ± 1.3	17.7 ± 1.2	17.4 ± 1.5		
	**BIPAP**_**AE**_	7.7 ± 0.6	17.8 ± 1.0	17.3 ± 1.9	18.1 ± 1.2	17.4 ± 0.7	17.6 ± 0.6		
Peak P_L_ (cmH_2_O)	**BIPAP**_**SB**_	6.4 ± 1.0	21.2 ± 1.4^e^	21.0 ± 1.4^e^	21.3 ± 1.1^e^	20.6 ± 2.0^e^	21.5 ± 1.8^e^	0.282	0.019
	**BIPAP**_**PC**_	6.8 ± 1.2	18.0 ± 1.2	17.5 ± 1.1	17.8 ± 1.7	17.5 ± 1.6	17.3 ± 1.8		
	**BIPAP**_**AI**_	6.7 ± 1.2	18.4 ± 1.4	17.3 ± 1.1	18.7 ± 1.3	17.3 ± 1.5	18.4 ± 1.2		
	**BIPAP**_**AE**_	7.0 ± 1.2	17.9 ± 1.8	17.5 ± 1.3	18.7 ± 1.8	18.8 ± 1.5	18.0 ± 1.2		
ΔPeso (cmH_2_O)	**BIPAP**_**SB**_	4.5 ± 0.6	13.5 ± 2.4^e^	12.6 ± 2.0^e^	13.5 ± 3.2^e^	12.4 ± 2.3^e^	13.2 ± 2.8^e^	0.456	0.01
	**BIPAP**_**PC**_	4.7 ± 0.6	4.5 ± 0.6^a,c^	4.5 ± 0.6^a,c^	3.7 ± 0.6^a,c^	3.9 ± 0.7^a,c^	4.3 ± 0.4^a,c^		
	**BIPAP**_**AI**_	4.3 ± 0.5	8.5 ± 0.7^e^	8.5 ± 0.7^e^	7.8 ± 0.7^e^	8.5 ± 0.8^e^	7.8 ± 1.0^e^		
	**BIPAP**_**AE**_	4.4 ± 0.8	4.5 ± 0.9^a,c^	3.8 ± 1.6^a,c^	3.9 ± 1.3^a,c^	4.3 ± 0.4^a,c^	3.9 ± 1.4^a,c^		
Pgas (cmH_2_O)	**BIPAP**_**SB**_	4.6 ± 2.1	13.0 ± 2.4^e^	12.3 ± 1.7^e^	13.5 ± 2.0^e^	12.1 ± 2.6^e^	12.5 ± 1.8^e^	0.476	0.002
	**BIPAP**_**PC**_	4.2 ± 2.0	5.2 ± 1.0	4.0 ± 0.6^e^	4.6 ± 0.9^e^	5.0 ± 0.6^a,d^	4.1 ± 0.9^e^		
	**BIPAP**_**AI**_	4.4 ± 2.3	5.8 ± 1.6	5.3 ± 1.4	5.7 ± 1.6	5.7 ± 1.0^a,d^	5.9 ± 0.8^e^		
	**BIPAP**_**AE**_	6.6 ± 1.8	6.0 ± 1.6	7.6 ± 1.8^b,c^	7.4 ± 1.6^b,c^	7.1 ± 0.6^b,c^	7.2 ± 1.3^e^		

Values are means ± SD. ^a^p < 0.05, compared with BIPAP_SB_ group; ^b^p < 0.05 compared with BIPAP_PC_ group; ^c^p < 0.05 compared with BIPAP_AI_ group; ^d^p < 0.05 compared with BIPAP_AE_ group; ^e^p < 0.05 compared with other groups. BIPAP_SB_ = biphasic positive airway pressure with SB; BIPAP_PC_ = biphasic positive airway pressure with muscles paralysis; BIPAP_AI_ = biphasic positive airway pressure with inspiratory muscles activity; BIPAP_AE_ = biphasic positive airway pressure with expiratory muscles activity; SB = spontaneous breathing; MV = minute ventilation; PaCO_2_ = partial pressure of carbon dioxide; PaO_2_/FiO_2_ = ratio of partial pressure of arterial oxygen to faction of inspired oxygen concentration; RR = respiratory rate; VTave = average tidal volume; P_plat_ = plateau pressure; PTP = pressure time product; mean Paw = mean airway pressure; peak P_L_ = peak transpulmonary pressure; mean P_L_ = mean transpulmonary pressure; Peso = esophageal pressure; ΔPes = change of esophageal pressure; Pgas = intragastric pressure.

**Table 2:** Histological sub-scores in experimental groups.

	BIPAP_SB_	BIPAP_PC_	BIPAP_AP_	BIPAP_PT_	F value	P value
Congestion	2.8 ± 0.5	3.2 ± 0.7	2.1 ± 0.4	3.3 ± 0.6	5.663	0.006
Edema, interstitial	2.4 ± 0.5	3.0 ± 0.4	2.2 ± 0.8	3.3 ± 0.3	0.497	0.689
Edema, alveolar	2.1 ± 0.7	3.3 ± 0.5	2.1 ± 0.5	3.3 ± 0.5	8.955	0.001
Granulocyte infiltrate, interstitial	2.5 ± 0.6	2.8 ± 0.6	2.2 ± 0.5	3.3 ± 0.2	4.152	0.019
Granulocyte infiltrate, alveolar	2.7 ± 0.6	2.9 ± 0.5	2.0 ± 0.6	3.2 ± 0.5	2.255	0.113
Erythrocyte infiltrate, interstitial	2.8 ± 0.4	2.8 ± 0.6	2.4 ± 0.6	3.2 ± 0.3	5.511	0.006
Erythrocyte infiltrate, alveolar	2.8 ± 0.6	2.9 ± 0.5	2.4 ± 0.8	3.2 ± 0.5	4.526	0.014
Lymphocyte infiltrate, interstitial	2.6 ± 0.5	3.0 ± 0.3	2.2 ± 0.7	3.0 ± 0.7	1.528	0.238
Microthrombi	2.2 ± 0.3	2.7 ± 0.4	2.3 ± 0.9	3.2 ± 0.3	1.935	0.156
Fibrinous exudate, interstitia	2.3 ± 0.5	2.5 ± 0.5	2.3 ± 0.5	3.4 ± 0.3	3.767	0.027
Fibrinous exudate, alveolar	2.2 ± 0.3	2.7 ± 0.5	2.3 ± 0.4	3.0 ± 0.4	1.77	0.185
Cumulative score	26.1 ± 2.1	29.2 ± 2.3	23.1 ± 2.1	32.4 ± 2.2	19.8	0.003

Values are means ± SD. BIPAP_SB_ = biphasic positive airway pressure with SB; BIPAP_PC_ = biphasic positive airway pressure with muscles paralysis; BIPAP_AI_ = biphasic positive airway pressure with inspiratory muscles activity; BIPAP_AE_ = biphasic positive airway pressure with expiratory muscles activity; SB = spontaneous breathing; Grading as: 0, minimal changes; 1, mild; 2, moderate; 3, severe; 4, maximal changes.
