# Supplementary material for: An experimental study on the impacts of inspiratory and expiratory muscles activities during mechanical ventilation in ARDS animal model
Source: Sci Rep. 2017 Feb 23;7:42785. doi: 10.1038/srep42785 (PMC5322359; doi:10.1038/srep42785)
Supplement: Supplementary Information [file srep42785-s1.pdf]

An experimental study on the impacts of inspiratory and expiratory muscles activities during mechanical ventilation in ARDS animal model

Xianming Zhang[1], Juan Du[1],Weiliang Wu[2], Yongcheng Zhu[2], Ying Jiang[2], Rongchang Chen[2]*

1. Department of Respiratory Medicine, First Affiliated Hospital of Guizhou Medical University, Guizhou, China

2. Respiratory Mechanics Lab, State Key Laboratory of Respiratory Disease, Guangzhou Institute of Respiratory Disease,

First Affiliated Hospital of Guangzhou Medical University, Guangzhou, Guangdong, China

Representative respiratory tracings of airway pressure(Paw), esophageal pressure (Pes) 、intragastric pressure （Pgas），transpumonary pressure（PL），Airflow，tidal volume，abdominal muscles surface electromyography （ EMGab ） and diaphragmatic esophageal surface electromyography（EMGdi) in $BIPAP_{SB}$、$BIPAP_{AI}$、$BIPAP_{PC}$ and $BIPAP_{AE}$ group in representative animals.

$BIPAP_{SB}$

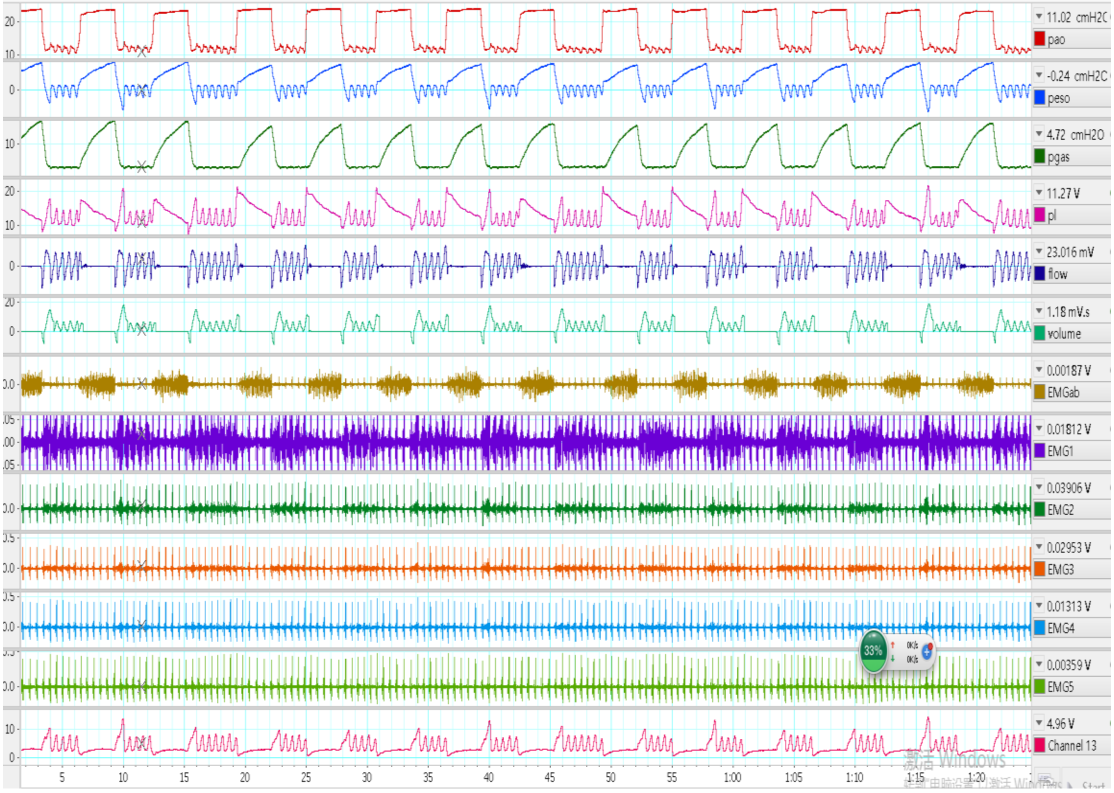

Representative respiratory tracings of airway pressure(Paw), esophageal pressure (Pes) 、intragastric pressure （Pgas），transpumonary pressure（PL），Airflow，tidal volume，abdominal muscles surface electromyography（EMGab），diaphragmatic esophageal surface electromyography（EMGdi)（1-5）and transdiaphragmatic pressure（Pdi） in $BIPAP_{SB}$ group in representative animals.

BIPAP$_{pc}$

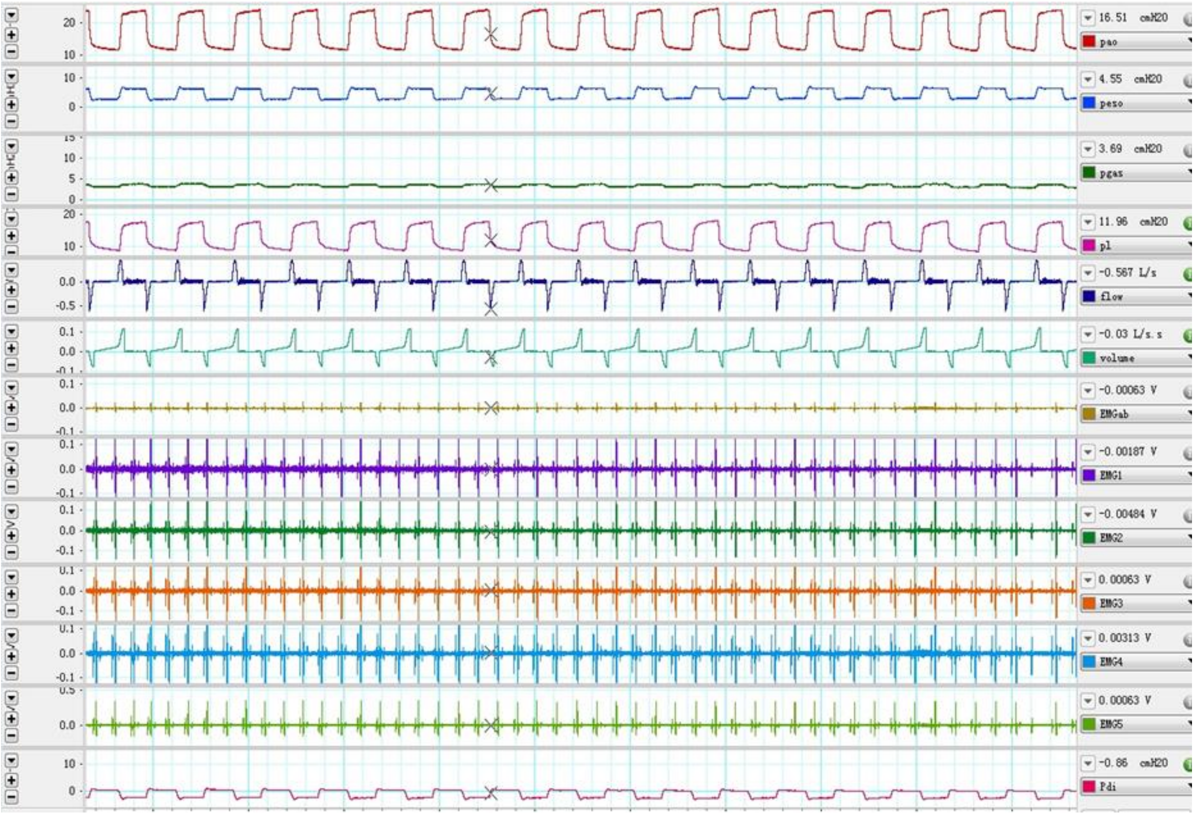

Representative respiratory tracings of airway pressure(Paw), esophageal pressure (Pes) 、intragastric pressure （Pgas），transpumonary pressure（PL），Airflow，tidal volume，abdominal muscles surface electromyography（EMGab），diaphragmatic esophageal surface electromyography（EMGdi)（1-5）and transdiaphragmatic pressure（Pdi） in BIPAP$_{PC}$ group in representative animals

BIPAP$_{AI}$

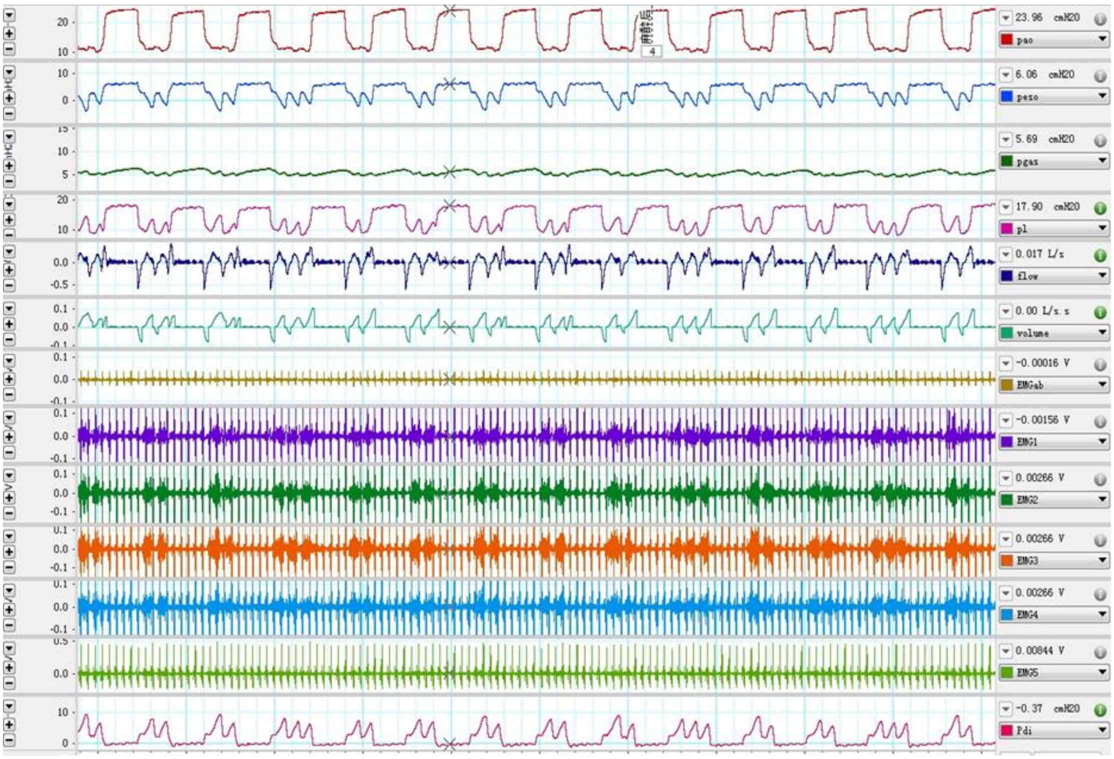

Representative respiratory tracings of airway pressure(Paw), esophageal pressure (Pes) 、intragastric pressure （Pgas），transpumonary pressure（PL），Airflow，tidal volume，abdominal muscles surface electromyography（EMGab），diaphragmatic esophageal surface electromyography（EMGdi)（1-5）and transdiaphragmatic pressure（Pdi） in BIPAP$_{AI}$ group in representative animals

BIPAP$_{AE}$

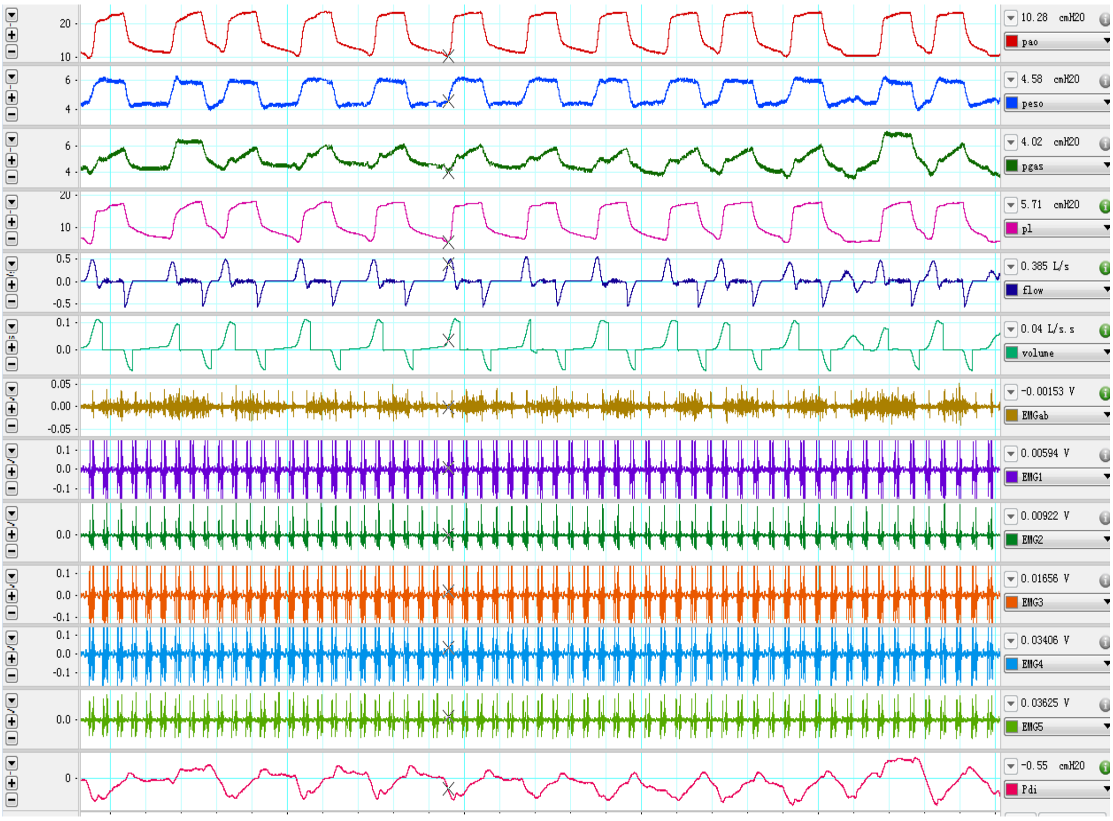

Representative respiratory tracings of airway pressure(Paw), esophageal pressure (Pes) 、intragastric pressure （Pgas），transpumonary pressure（PL），Airflow，tidal volume，abdominal muscles surface electromyography（EMGab），diaphragmatic esophageal surface electromyography （EMGdi)（1-5）and transdiaphragmatic pressure（Pdi） in BIPAP$_{AE}$ group in representative animals
